# Complete genome sequence of *Imperialibacter roseus* strain P4^T^

**DOI:** 10.1128/mra.00986-23

**Published:** 2024-05-23

**Authors:** Daniela Tizabi, Tsvetan Bachvaroff, Russell T. Hill

**Affiliations:** 1 Institute of Marine and Environmental Technology, University of Maryland Center for Environmental Science, Baltimore, Maryland, USA; California State University San Marcos, San Marcos, California, USA

**Keywords:** *Imperialibacter roseus*

## Abstract

*Imperialibacter roseus* strain P4^T^ is a bacterial strain isolated from Permian groundwater. The complete genome of *Imperialibacter roseus* strain P4^T^ was sequenced to reveal a single circular chromosome of 6,747,663 bp with a GC content of 46.5%.

## ANNOUNCEMENT

*Imperialibacter roseus* strain P4^T^ was isolated from Permian groundwater in Imperial, Texas (31° 16′ 16.93″ N 102° 40′ 48.35″ W) in 2011 (1). As the representative species of this recently designated genus, sequencing was performed to better characterize *Imperialibacter* spp. This Gram-negative bacterium is aerobic, rod-shaped, and belongs to the family *Flammeovirgaceae* (1). The groundwater collected from Pecos Cenozoic Trough retains the chemical composition of an ancient ocean, with significantly higher concentrations of HCO_3_^-^, Ca^2+^, NO_3_^-^, and SiO_3_^-^, and a reduced salinity of 13 ppt (2). *Imperialibacter roseus* strain P4^T^ was characterized by polyphasic analysis (1).

*Imperialibacter roseus* strain P4^T^ [originally isolated on Difco marine agar 2216 at 30°C and preserved at −80°C in marine broth 2216, supplemented with 30% (vol/vol) glycerol] was grown on fresh marine agar 2216 at 30°C until visible colonies formed. Although original isolates presented as pink colonies, recent cultivation produces orange colonies. DNA extraction of two colonies was performed with the UltraClean Microbial DNA Isolation Kit. A sequencing library was prepared using the Native Barcoding Kit 24 V14 (SQK-NBD114.24, Oxford Nanopore Technologies) with no DNA size selection, and sequenced with an R10.4.1 flow cell on a MinION device at 400 bases per second. Base calling was performed with Dorado (model dna_r10.4.1_e8.2_400bps_sup@v4.2.0), and reads were demultiplexed with Porechop v.0.2.4. Read quality was assessed with FastQC v.0.12.0 (3), and 1,446,827 reads were originally generated ranging from 2 to 345,629 bases. A size-filtered subset of the raw reads ≥8 kb (134,148 reads) was used for assembly with Flye v.2.9.1-b1780 (4, 5) to reduce coverage and generate one contig, circularized with Circlator v.1.5.5 (6). The genome assembled to 6.7 Mb with 46.5% GC content and 202× coverage. Annotation with the NCBI Prokaryotic Genome Annotation Pipeline revealed 5,692 genes, 5,628 coding sequences, 40 tRNAs, and 6 rRNAs. Two identical 16S ribosomal RNA gene copies were present, with a 92% query cover and 99.65% identity to *Imperialibacter roseus* strain P4 16S ribosomal RNA, partial sequence (GenBank accession no. NR_132295.1).

Only three other genomes are listed in NCBI from cultured *Imperialibacter* isolates (*Imperialibacter* spp. EC-SDR9, 75, and 89), all associated with the brown macroalgae *Ectocarpus subulatus* (BioProject accession nos. PRJEB22665, PRJEB31339) (7). All three isolates are assembled to the draft genome level with a size of 6.8 Mb with 65 to 73 scaffolds, consistent with *I. roseus* strain P4^T^. Although it is unclear whether the *E. subulatus* samples originated from France or Australia, it is interesting to find this bacterium reported in disparate and geographically isolated habitats. A bacterium with greater than 99% 16S rRNA identity to *Imperialibacter roseus* strain P4^T^ was recently identified in metagenomic sequencing data from a mixed culture of the dinoflagellate *Karlodinium veneficum* and obligate parasite *Amoebophyra* sp. ex *Karlodinium veneficum* grown from the Chesapeake Bay ( D. Tizabi, T. Bachvaroff, and R. Hill, unpublished data). Although attempts to culture this strain of *Imperialibacter* sp. have failed thus far, this observation further expands our knowledge of the geographic extent of this genus.

## Data Availability

The complete genome sequence has been deposited at DDBJ/ENA/GenBank under accession no. CP136051, BioSample accession no. SAMN37629620 and BioProject accession no. PRJNA1022829. A size-filtered subset of raw sequence reads 8kb has been deposited in the Sequence Read Archive (SRA) under accession no. SRR26278781.
